# Japanese Encephalitis, Tibet, China

**DOI:** 10.3201/eid1705.101417

**Published:** 2011-05

**Authors:** Yi-Xing Li, Ming-Hua Li, Shi-Hong Fu, Wei-Xin Chen, Qi-Yong Liu, Hai-Lin Zhang, Wa Da, Song-Lin Hu, Sang Dan La Mu, Ju Bai, Zun-Dong Yin, Hong-Yue Jiang, Yu-Hong Guo, Dun Zhu Duo Ji, Hui-Mei Xu, Ge Li, Gu Gu Cuo Mu, Hui-Ming Luo, Jing-Lin Wang, Jun Wang, Xiu-Min Ye, Zhuo Ma Yang Jin, Wei Zhang, Gui-Jun Ning, Huan-Yu Wang, Gui-Chang Li, Jian Yong, Xiao-Feng Liang, Guo-Dong Liang

**Affiliations:** Author affiliations: National Immunization Programme, Chinese Center for Disease Control and Prevention, Beijing, People’s Republic of China (Y.-X. Li, Z.-D. Yin, H.-M. Luo, G.-J. Ning, X.-F. Liang);; Institute for Viral Disease Control and Prevention, Beijing (M.-H. Li, S.-H. Fu, W.-X. Chen, H.-Y. Jiang, J.-L. Wang, H.-Y. Wang, G.-D. Liang);; National Institute for Communicable Disease Control and Prevention, Beijing (Q.-Y. Liu, Y.-H. Guo, J. Wang, G.-C. Li);; Yunnan Institute of Endemic Disease Control and Prevention, Yunnan, People’s Republic of China (H.-L. Zhang);; Tibet Center for Disease Control and Prevention, Tibet, People’s Republic of China (W. Da, D.Z.D. Ji, X.-M. Ye);; Nyingchi Center for Disease Control and Prevention, Tibet (S.-L. Hu, H.-M. Xu, Z.M.Y. Jin);; Medog County Center for Disease Control and Prevention, Tibet, (S.D.L. Mu, G. Li, W. Zhang, J. Yong);; Mainling County Center for Disease Control and Prevention, Tibet (J. Bai, G.G.C. Mu)

**Keywords:** Japanese encephalitis virus, isolation, identification, mosquitoes, viruses, vector-borne infections, Tibet, People’s Republic of China, letter

**To the Editor:** Tibet is located in the Qinghai-Tibet Plateau of western People’s Republic of China and has been internationally recognized as a Japanese encephalitis (JE)–nonendemic area because the average altitude is thought to be too high to facilitate the cycle of Japanese encephalitis virus (JEV) between mosquitoes and vertebrates (*1**,**2*). In addition, JE is a reportable infectious disease in China, and no clinically confirmed case has been reported in Tibet since establishment of a national case reporting system in 1951 (*3**,**4*). Neither the mosquito vector of JEV nor JEV isolates have been described in Tibet. In this study, JEV was isolated from *Culex tritaeniorhynchus* mosquitoes, the main vectors of JEV, collected in Tibet. Serologic assays detected anti-JEV antibodies in a large number of human and porcine serum samples collected in this region. These data demonstrate that JEV is currently circulating in Tibet.

During August 5–15, 2009, mosquitoes were collected in Mainling County (altitude 2,900 m) and Medog County (altitude 1,000 m) in the Nyingchi area of Tibet. A total of 4,089 mosquitoes representing 7 species (*Cx. tritaeniorhynchus, Cx. pipiens pallens, Cx. bitaeniorhynchus, Armigeres obturbans, Anopheles maculatus maculates, An. peditaeniatu*, and *Aedes albopictus*) from 4 genera were collected in this study. The dominant mosquito species detected in Medog County was *Cx. tritaeniorhynchus* (2,442 [71.1%] of 3,436 mosquitoes collected there) (Table); no previous reports have described this species in Tibet. A total of 653 mosquitoes were collected in Mainling County, of which 489 (74.9%) were *Armigeres obturban*. No *Cx. tritaeniorhynchus* mosquitoes were collected in Mainling County.

**Table:** Results from testing of mosquitoes, humans, and pigs for JEV, Nyingchi area, Tibet, People’s Republic of China, 2009*

Collection sites	Mosquitoes			Humans			Pigs	
	No. collected	*Culex tritaeniorhynchus*, no. (%)		No. samples	Neutralizing antibody titers against JEV >10, no. (%)		No. samples	JEV IgM antibody positive, no. (%)
Mainling County	653	0		100	22 (22.0)		30	17 (56.7)
Medog County	3,436	2,442 (71.1)†		148	46 (31.1)		36	5 (13.9)
Total	4,089	2,442 (59.7)		248	68 (27.4)		66	22 (33.3)

*JEV, Japanese encephalitis virus; Ig, immunoglobulin. †Pool was positive for JEV by PCR.

Mosquitoes were homogenized in 97 pools by using TissueLyser (QIAGEN, Hilden, Germany) and screened with reverse transcription–PCR (RT-PCR) by using seminested primers designed to detect the JEV *PreM* gene (*5*). One *Cx. tritaeniorhynchus* pool, XZ0938, collected in Medog County was positive by PCR. Isolation of virus was conducted from PCR-positive sample by injecting mosquito homogenate supernatants into monolayers of BHK-21 and C6/36 cells. The supernatant of pool XZ0938 caused cytopathic effects in BHK-21 and C6/36 cells in successive cell passages. The complete genome of 10,965 nt was sequenced (GenBank accession no. HQ652538) as described (*6*), which included a 96-nt 5′ nontranslated region and a 570-nt 3′ nontranslated region. The single open reading frame coded for a polyprotein of 3,432 aa. Compared with the complete genome sequences of 62 known JEV isolates, the nucleotide sequence identity varied from 83.6% to 97.8% and amino acid sequence identity from 94.9% to 99.7%. Phylogenetic trees derived from nucleotide sequences of the complete genome of JEV strains indicated that XZ0938 was a member of genotype I JEV. A more detailed analysis indicated that the Tibet JEV is most closely related to JEV isolates KV1899 (1999, Korea, AY316157), and JEV/sw/Mie/41/2002 (2002, Japan, AB241119) (data not shown).

To determine whether local residents were infected by JEV, 248 human serum samples were collected in Mainling and Medog Counties from healthy persons. Neutralizing antibody against JEV was tested by 90% plaque-reduction neutralization tests by using standard methods (*7*). Serum samples were tested with serial 2-fold dilutions from 1 to 5. Diluted serum was mixed with equal volumes of culture medium containing JEV P3 strain. The samples were considered positive when the neutralizing antibody titers >10. Sixty-eight positive samples were determined by 90% plaque-reduction neutralization tests, which constituted 68 (27.4%) of all 248 serum samples. Twenty-two (22.0%) of 100 and 46 (31.1%) of 148 serum samples in Mainling and Medog Counties, respectively, were positive (Table). Currently, the local population is not vaccinated against JEV (*8*) because Tibet is considered a JE-nonendemic area (*1**–**4*). The observation that 68 (27.4%) of 248 serum samples from healthy humans contained neutralizing antibody against JEV at titers >10 suggests that this population is subject to substantial levels of subclinical JEV infection.

To determine the present situation of JEV infection in local pigs, we analyzed 66 serum samples collected from piglets 1–6 months of age in Mainling and Medog Counties; immunoglobulin M antibodies against JEV were detected by capture ELISA as described (*9*). That 22 (33.3%) of 66 piglet serum samples were positive for immunoglobulin M against JEV suggested that local pigs have been newly infected by JEV in 2009 and have participated in the cycles of JEV in the local area (Table).

JE is a global public health issue that has spread to >20 countries in Asia (*6**,**10*). In this study, we present evidence that JEV has extended its geographic range to Tibet, a region that previously was believed to be free of JE because of its elevation. Factors such as global warming, increased pig farming, and increased tourism and transportation may have contributed to the emergence of JE in Tibet. Conditions in Tibet, including the presence of the primary vector (*Cx. tritaeniorhynchus* mosquitoes), abundant amplification hosts (pig), and a naive population that has not been vaccinated against JEV, present the possibility for JE outbreaks. Increased surveillance for JE in this region is needed.
